# Lobe‐Specific Versus Systematic Lymph Node Dissection in Clinical Stage I Non‐Small Cell Lung Cancer: A Propensity Score‐Matched Analysis Based on the 8th Edition of the TNM Stage Classification

**DOI:** 10.1111/1759-7714.70337

**Published:** 2026-06-29

**Authors:** Hirotsugu Notsuda, Ken Onodera, Sakiko Kumata, Satoshi Kamata, Toru Kawakami, Tatsuaki Watanabe, Yui Watanabe, Takashi Hirama, Takaya Suzuki, Hisashi Oishi, Hiromichi Niikawa, Yoshinori Okada

**Affiliations:** ^1^ Department of Thoracic Surgery Tohoku University Hospital Sendai Miyagi Japan; ^2^ Department of Thoracic Surgery, Institute of Development, Aging and Cancer Tohoku University Sendai Miyagi Japan; ^3^ Department of Thoracic Surgery Tohoku Medical and Pharmaceutical University Sendai Miyagi Japan

## Abstract

**Background:**

The optimal extent of lymph node dissection (LND) in early‐stage non–small cell lung cancer (NSCLC) remains controversial. This study aimed to compare perioperative and oncological outcomes between lobe‐specific LND (LSD) and systematic LND (SND) in patients with clinical stage I NSCLC based on the 8th edition of the TNM classification.

**Methods:**

We retrospectively analyzed 390 patients with clinical stage I NSCLC who underwent lobectomy with LND between 2010 and 2020. Patients with tumors in the right middle lobe or left lingular segment were excluded. Propensity score matching (PSM; 1:1 nearest‐neighbor matching) was performed using clinical and radiological covariates, yielding 140 matched patients in each group. Perioperative outcomes, postoperative complications (Clavien–Dindo grade ≥ II, with postoperative pneumonitis assessed according to CTCAE version 5.0), overall survival (OS), relapse‐free survival (RFS), and recurrence patterns were compared.

**Results:**

After PSM, the LSD group had significantly shorter operative time, less blood loss, and shorter durations of chest tube placement and postoperative hospital stay than the SND group (all *p* < 0.05). Postoperative supraventricular tachyarrhythmia occurred more frequently in the SND group (0.7% vs. 7.1%, *p* = 0.006), whereas other complications were comparable. There were no significant differences in OS or RFS before or after PSM. Recurrence rates and patterns, including ipsilateral lymph node recurrence, were comparable.

**Conclusions:**

LSD appears to be an oncologically valid and less invasive alternative to SND in patients with clinical stage I NSCLC. LSD may reduce surgical invasiveness and postoperative morbidity without compromising survival or increasing the risk of recurrence.

## Graphical abstract

In this propensity score‐matched study of clinical stage I non‐small cell lung cancer, lobe‐specific lymph node dissection achieved comparable overall and relapse‐free survival to systematic lymph node dissection while reducing operative invasiveness and postoperative supraventricular tachyarrhythmia.

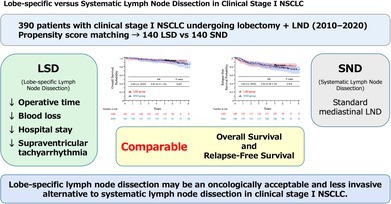

**Abbreviations**
%FEV1.0: percent predicted forced expiratory volume in 1 s
CCI: Charlson Comorbidity Index
CEA: carcinoembryonic antigen
CT: computed tomography
DFS: disease‐free survival
GGN: ground‐glass nodule
LN: lymph node
LND: lymph node dissection
LSD: lobe‐specific lymph node dissection
NSCLC: non‐small cell lung cancer
OS: overall survival
RFS: relapse‐free survival
SND: systematic lymph node dissection

## 1 Introduction

Lymph node dissection (LND) is an essential component of the surgical treatment for non‐small cell lung cancer (NSCLC), enabling accurate pathological staging and the establishment of appropriate postoperative management strategies [1, 2]. Systematic lymph node dissection (SND), which involves the comprehensive removal of hilar and mediastinal lymph nodes (LNs), has long been considered the standard procedure performed in conjunction with lobectomy for NSCLC [3, 4, 5]. However, in patients with early‐stage NSCLC, lobe‐specific lymph node dissection (LSD) has emerged as a less invasive alternative to SND, whereby the extent of mediastinal dissection is selectively tailored according to the tumor location [6, 7]. This approach is based on accumulating evidence demonstrating that lymphatic spread in NSCLC follows lobe‐specific patterns [8]. Metastasis from upper‐lobe cancers to the subcarinal LNs is uncommon, whereas lower‐lobe cancers rarely metastasize to the upper mediastinal LNs [9, 10, 11, 12]. Several retrospective studies have reported no significant differences in survival outcomes between patients undergoing LSD and those undergoing SND [13, 14, 15], suggesting that LSD may be an oncologically acceptable alternative. Therefore, a significant proportion of institutions have already adopted LSD as a standard practice for radical treatment in patients with early‐stage NSCLC. However, evidence supporting the oncological non‐inferiority of LSD compared with SND remains limited, particularly under the 8th edition of the TNM classification, and independent validation studies from institutions other than large‐volume centers are scarce [16].

In 2023, a large‐scale cohort study from the National Cancer Center Hospital East reported that overall survival (OS) and relapse‐free survival (RFS) did not differ significantly between patients with clinical stage I NSCLC who underwent SND and those who underwent LSD based on the 8th edition of the TNM stage classification [17]. Baseline characteristics between the two groups were adjusted using propensity score matching (PSM) in this study. At the same time, the authors demonstrated that patients with clinical stage II disease in the LSD group tended to have worse OS and RFS than those in the SND group [17]. This study has become an important reference in selecting patients appropriate for LND; however, independent validation by other institutions remains insufficient.

At our institution, the extent of LND for clinical stage I NSCLC has evolved over time in accordance with institutional policy, informed by the literature supporting the oncological non‐inferiority of LSD compared with SND. SND was routinely performed until 2015, after which LSD was adopted for patients with clinical stage I disease. More than a decade after this strategic shift in surgical practice, we sought to evaluate and compare the impact of these two surgical procedures on perioperative and oncological outcomes in patients with clinical stage I NSCLC. While this comparative approach has the inherent limitation that the two techniques were performed during different time periods, it has an important advantage: the choice of surgical procedures was determined uniformly by institutional policy rather than by individual surgeon preference, thereby minimizing patient selection bias. In addition, we applied PSM to further adjust for potential confounding factors between the two groups in the present study.

The aim of this study was to compare surgical and postoperative outcomes, survival, and recurrence patterns between LSD and SND in patients with clinical stage I NSCLC according to the 8th edition of the TNM classification.

## 2 Methods

The Ethics Committee of Tohoku University Graduate School of Medicine approved this retrospective study (No. 2021‐1‐912‐1) and waived the requirement for written informed consent.

### 2.1 Patient Eligibility

This retrospective cohort study was conducted at a single institution. Patients who underwent surgical resection for suspected or confirmed NSCLC between 2010 and 2020 were screened for eligibility. Inclusion criteria were clinical stage I NSCLC and complete resection by lobectomy with LND. Exclusion criteria included patients who underwent induction therapy, those who underwent a surgical procedure other than lobectomy, those pathologically diagnosed with disease other than NSCLC, and those with tumors located in the right middle lobe or left lingular segment.

### 2.2 Preoperative Staging

Preoperative staging was performed using contrast‐enhanced computed tomography (CT) and 18F‐fluorodeoxyglucose positron emission tomography/CT (FDG‐PET/CT). Contrast‐enhanced CT scans with 5–10 mm collimation of the chest and upper abdomen were obtained for clinical staging. In addition, thin‐section CT images with 1–2 mm collimation were reconstructed to evaluate tumor size, ground‐glass opacity component, and solid tumor size. Lymph node involvement was suspected when mediastinal lymph nodes exceeded 1.0 cm in the short‐axis diameter on CT and/or showed abnormal FDG uptake on PET/CT. When mediastinal lymph node metastasis was radiologically suspected, endobronchial ultrasound‐guided transbronchial needle aspiration (EBUS‐TBNA) was performed for pathological confirmation and to determine surgical indication.

### 2.3 Surgical Procedure

All patients underwent lobectomy with dissection of the hilar and mediastinal LNs. Dissected LNs were classified according to their anatomical location based on the International Association for the Study of Lung Cancer (IASLC) nodal map [18]. According to the extent of mediastinal lymph node dissection, patients were categorized into two groups: those who underwent SND and those who underwent LSD. SND was routinely performed until 2015, whereas LSD was introduced thereafter for patients with clinical stage I NSCLC.

The extent of lymph node dissection according to tumor location is summarized in Table S1. For upper‐lobe tumors, upper mediastinal lymph node dissection was routinely performed, whereas subcarinal lymph node dissection was omitted. In contrast, for lower‐lobe tumors, subcarinal lymph node dissection was routinely performed, whereas upper mediastinal lymph node dissection was omitted. In both approaches, hilar (#10), interlobar (#11), and peripheral zone nodes (#12–14) were routinely dissected.

First recurrence sites were classified as ipsilateral LN recurrence or other sites, which included ipsilateral pulmonary metastasis, pleural effusion, pleural dissemination, and distant metastasis.

### 2.4 Data Collection

Data were obtained from our institutional NSCLC database. Collected variables included age, sex, smoking index, percent predicted forced expiratory volume in 1 s (%FEV1.0), tumor size, radiological pattern (solid, part‐solid or pure solid), solid tumor size on computed tomography, clinical pleural invasion, carcinoembryonic antigen (CEA), histological subtype according to the World Health Organization classification, and stage according to the 8th edition of the TNM classification. Comorbidity status was evaluated using the age‐adjusted Charlson Comorbidity Index. Postoperative complications were primarily classified according to the Clavien–Dindo classification, and complications of grade II or higher were included in the analysis. Postoperative arrhythmia was defined as supraventricular tachyarrhythmia diagnosed during the postoperative hospital stay. Further subclassification of arrhythmia (e.g., atrial fibrillation or paroxysmal atrial tachycardia) was not feasible due to limitations of retrospective data collection. Postoperative pneumonitis was assessed separately and defined according to the Common Terminology Criteria for Adverse Events (CTCAE), version 5.0. Patients were followed up every 3 to 12 months for at least 5 years.

### 2.5 Statistical Analyses

Differences between groups for categorical variables were assessed using the chi‐square test or Fisher's exact test. An unpaired *t*‐test was used for comparisons of normally distributed variables, while the Mann–Whitney *U* test was used for non‐normally distributed variables. Survival curves were generated using the Kaplan–Meier method, and intergroup differences were evaluated with the log‐rank test. OS was defined as the interval from the date of surgery to the date of death or last follow‐up. RFS was defined as the interval from the date of surgery to the date of first recurrence, death, or last follow‐up. As a sensitivity analysis to account for differences in follow‐up duration between the groups, restricted mean survival time (RMST) was calculated up to 60 months after surgery and compared between the LSD and SND groups.

OS and RFS were compared between the two groups both before and after PSM. A two‐sided *p*‐value of less than 0.05 was considered statistically significant. Propensity scores were estimated using a logistic regression model and covariates included age, sex, smoking index, percent predicted forced expiratory volume in 1 s (%FEV1.0), Charlson Comorbidity Index, nodule location, solid tumor size on CT, radiological pattern, clinical pleural invasion, carcinoembryonic antigen, clinical stage, and histology. Nearest‐neighbor 1:1 matching without replacement was performed. Covariate balance before and after PSM was assessed using standardized differences, with an absolute value of less than 0.1 indicating adequate balance. There were no substantial missing data in the variables used for propensity score estimation. All statistical analyses were performed using JMP Student Edition 19 (JMP Statistical Discovery LLC, Cary, NC, USA).

## 3 Results

### 3.1 Patient Characteristics

**FIGURE 1:**
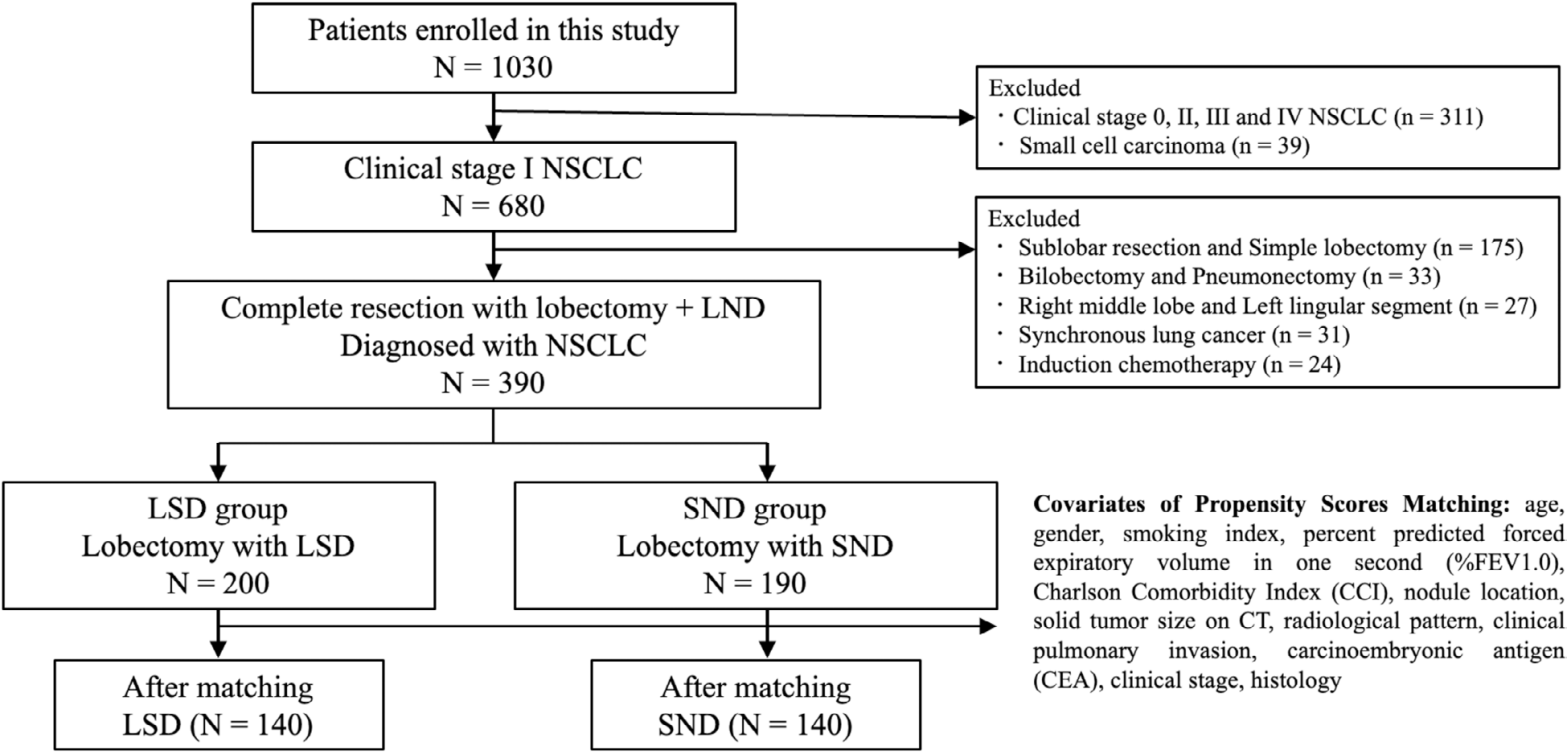
Flow diagram of patient selection and propensity score matching. Between 2010 and 2020, a total of 1030 patients underwent surgical resection for suspected or confirmed non‐small cell lung cancer (NSCLC) at our institution. Patients who underwent sublobar resection or simple lobectomy without mediastinal lymph node dissection were excluded. After applying the exclusion criteria, 390 patients with clinical stage I NSCLC who underwent complete resection with lobectomy and lymph node dissection (LND) were included. Patients were classified into the lobe‐specific lymph node dissection (LSD) group and the systematic lymph node dissection (SND) group. Propensity score matching was performed using predefined covariates, resulting in 140 matched patients in each group.

A total of 1030 consecutive patients underwent surgical resection for suspected or confirmed NSCLC during the study period. After applying the inclusion and exclusion criteria, 390 patients with clinical stage I NSCLC who underwent complete resection with lobectomy and LND were included in the final analysis (Figure 1). Patient characteristics of the LSD and SND groups before and after PSM are summarized in Tables 1 and 2, respectively. Before PSM, there were no significant differences in age, sex, smoking index, %FEV1.0, nodule location, CEA level, CT findings, or histological subtype between the two groups. However, the LSD group had a higher Charlson Comorbidity Index (*p* = 0.01), a higher proportion of clinical pleural invasion (*p* = 0.003), a smaller solid tumor size on CT (*p* < 0.001), and a lower clinical stage than the SND group (p < 0.001). Regarding pathological upstaging to stage III disease in the SND group, only one patient showed nodal metastasis exclusively outside the lobe‐specific dissection field; however, this patient also had concurrent metastasis within the LSD field. Therefore, no cases were identified in which pathological staging would have differed if LSD had been performed. After PSM, 140 patients were included in each group, and patient characteristics were well balanced, ensuring comparability for subsequent analyses.

**TABLE 1:** Comparison of patient characteristics between the LSD and SND groups.

Variables		LSD *N* = 200 Frequency (%)	SND *N* = 190 Frequency (%)	p	Standardized difference
Age	Median (IQR)	69 (62–73)	69 (64–74)	0.134	0.15
Gender	Female	92 (46.0)	92 (48.4)	0.632	0.04
Smoking index	≦ 40	87 (43.5)	84 (44.2)	0.888	0.01
%FEV1.0	< 70%	43 (21.5)	51 (26.8)	0.2176	0.12
CCI	0–2	33 (16.5)	21 (11.1)	0.01	0.16
	3–4	127 (63.5)	147 (77.4)		0.31
	5 ≦	40 (20.0)	22 (11.6)		0.22
Nodule location	Right upper lobe	85 (42.4)	75 (39.5)	0.278	0.06
	Right lower lobe	44 (22.0)	33 (17.4)		0.12
	Left upper lobe	32 (16.0)	44 (23.2)		0.18
	Left lower lobe	39 (19.5)	38 (20.0)		0.01
CEA	≧ 5 ng/mL	36 (18.0)	48 (25.3)	0.081	0.18
CT findings	Part‐solid GGN	87 (43.5)	84 (44.2)	0.888	0.02
	Solid	113 (56.5)	106 (55.8)		
Clinical pleural invasion	Yes	18 (9.0)	37 (19.5)	0.003	0.3
Solid tumor size on CT (cm)	Median (IQR)	1.7 (12–24)	2.3 (15–28)	< 0.001	0.42
Clinical stage	IA1	37 (18.5)	16 (8.4)	< 0.001	0.3
	IA2	75 (37.5)	57 (30)		0.16
	IA3	54 (27.0)	58 (30.5)		0.07
	IB	34 (17.0)	59 (31.1)		0.33
Histology	Adenocarcinoma	172 (86.0)	156 (82.1)	0.894	0.11
	Squamous cell carcinoma	19 (9.5)	29 (15.3)		0.17
	Others	9 (4.5)	5 (2.6)		0.1
Pathological stage	IA	147 (73.5)	117 (61.6)	0.063	
	IB	30 (15.0)	44 (23.2)		
	II	14 (7.0)	14 (7.4)		
	III	9 (4.5)	15 (7.9)		
Adjuvant Chemotherapy	Yes	44 (22.0)	45 (23.7)	0.692	

Abbreviations: %FEV1.0, percent predicted forced expiratory volume in 1 s; CCI, Charlson comorbidity index; CEA, carcinoembryonic antigen; CT, computed tomography; GGN, ground‑glass nodule; IQR, interquartile range; LSD, lobe‑specific nodal dissection; SND, systematic lymph node dissection.

**TABLE 2:** Comparison of patient characteristics between the LSD and SND groups after propensity score matching.

Variables		LSD *N* = 140 Frequency (%)	SND *N* = 140 Frequency (%)	*p*	Standardized difference
Age	Median (IQR)	69 (64–73)	68 (63–74)	0.776	0.04
Gender	Female	68 (48.6)	70 (50.0)	0.811	0.02
Smoking index	≦ 40	65 (46.4)	64 (45.7)	0.904	0.01
%FEV1.0	< 70%	37 (26.4)	30 (21.4)	0.327	0.09
CCI	0–2	16 (11.4)	18 (12.9)	0.386	0.03
	3–4	110 (78.6)	111 (79.3)		0.02
	5≦	14 (10.0)	11 (7.9)		0.08
Nodule location	Right upper lobe	57 (40.7)	57 (40.7)	0.8232	0
	Right lower lobe	28 (20.0)	28 (20.0)		0
	Left upper lobe	25 (17.9)	30 (21.4)		0.08
	Left lower lobe	30 (21.4)	25 (17.9)		0.08
CEA	≧5 ng/ml	28 (20.0)	29 (20.7)	0.882	0.03
CT findings	Part‐solid GGN	60 (42.9)	64 (45.7)	0.63	0.04
	Solid	80 (57.1)	76 (54.3)		
Clinical pleural invasion	Yes	15 (10.7)	18 (12.9)	0.578	0.06
Solid tumor size on CT (cm)	Median (IQR)	2.0 (1.3–2.6)	2.0 (1.5–2.7)	0.656	0.01
Clinical stage	IA1	18 (12.6)	16 (11.4)	0.9833	0.03
	IA2	49 (35.0)	49 (35.0)		0
	IA3	43 (30.7)	45 (32.1)		0.04
	IB	30 (21.4)	30 (21.4)		0
Histology	Adenocarcinoma	116 (82.9)	121 (86.4)	0.7092	0.08
	Squamous cell carcinoma	19 (13.6)	15 (10.7)		0.09
	Others	5 (3.6)	4 (2.9)		0.04
Pathological stage	IA	97 (69.3)	90 (64.3)	0.559	
	IB	23 (16.4)	27 (19.3)		
	II	13 (9.3)	11 (7.9)		
	III	7 (5.0)	12 (8.6)		
Adjuvant chemotherapy	Yes	35 (25.0)	37 (26.4)	0.784	

Abbreviations: %FEV1.0, percent predicted forced expiratory volume in 1 s; CCI, Charlson comorbidity index; CEA, carcinoembryonic antigen; CT, computed tomography; GGN, ground‑glass nodule; IQR, interquartile range; LSD, lobe‑specific nodal dissection; SND, systematic lymph node dissection.

### 3.2 Surgical and Postoperative Outcomes

Surgical and postoperative outcomes after PSM are shown in Table 3. The SND group had a significantly longer operative time (*p* < 0.001), greater blood loss (*p* = 0.036), longer durations of chest tube placement (*p* = 0.028), and postoperative hospital stay (*p* < 0.001) compared with the LSD group. Similar trends were observed before PSM, with the SND group demonstrating longer operative time, greater blood loss, and longer durations of chest tube placement and postoperative hospital stay compared with the LSD group (Table S2). Because of the chronological transition in our institutional surgical practice, almost all patients in the LSD group underwent VATS, whereas most patients in the SND group underwent open thoracotomy. Therefore, the surgical approach was strongly associated with the study period and could not be adequately balanced between the groups.

**TABLE 3:** Comparison of surgical and postoperative outcomes between the LSD and SND groups after propensity score matching.

Variables	LSD *N* = 140 Median (IQR)	SND *N* = 140 Median (IQR)	*p*
Operative time (min)	175 (144–212)	222 (188–255)	< 0.001
Blood loss (mL)	10 (4–40)	32 (10–83)	0.036
Chest tube placement (days)	2 (2–3)	3 (2–4)	0.028
Postoperative length of hospital stay (days)	8 (7–10)	11 (10–14)	< 0.001

Abbreviations: LSD, lobe‐specific nodal dissection; SND, systematic lymph node dissection.

### 3.3 Postoperative Complications

Postoperative complications after PSM are summarized in Table 4. Postoperative pneumonitis was evaluated based on CTCAE version 5.0, whereas other postoperative complications were classified according to the Clavien–Dindo system. The incidence of postoperative supraventricular tachyarrhythmia was significantly higher in the SND group than in the LSD group (*p* = 0.006). Notably, all cases of postoperative supraventricular tachyarrhythmia occurred in patients who underwent upper mediastinal LN dissection regardless of the LND or SND group. There were no differences in other complications including pneumonia and postoperative air leak lasting ≧ 7 days. Before PSM, the overall incidence of postoperative complications and arrhythmia was also higher in the SND group compared with the LSD group (*p* = 0.049 and *p* < 0.001, respectively) (Table S3).

**TABLE 4:** Comparison of ^a^postoperative complications between LSD and SND groups after propensity score matching.

Variables	LSD *N* = 140 Frequency (%)	SND *N* = 140 Frequency (%)	*p*
Postoperative complications	22 (15.7)	28 (20.0)	0.349
Supraventricular tachyarrhythmia	1 (0.7)	10 (7.1)	0.006
Pneumonitis/Atelectasis	4 (2.9)	5 (3.6)	0.735
Postoperative air leakage ≧ 7 days	11 (7.9)	12 (8.6)	0.828
Others	8 (5.7)	10 (7.1)	0.626

Abbreviations: LSD, lobe‐specific nodal dissection; SND, systematic lymph node dissection.

^a^ Postoperative complications were defined as Clavien–Dindo grade II or higher, except for postoperative pneumonitis, which was assessed according to the Common Terminology Criteria for Adverse Events (CTCAE), version 5.0.

### 3.4 Survival

**FIGURE 2:**
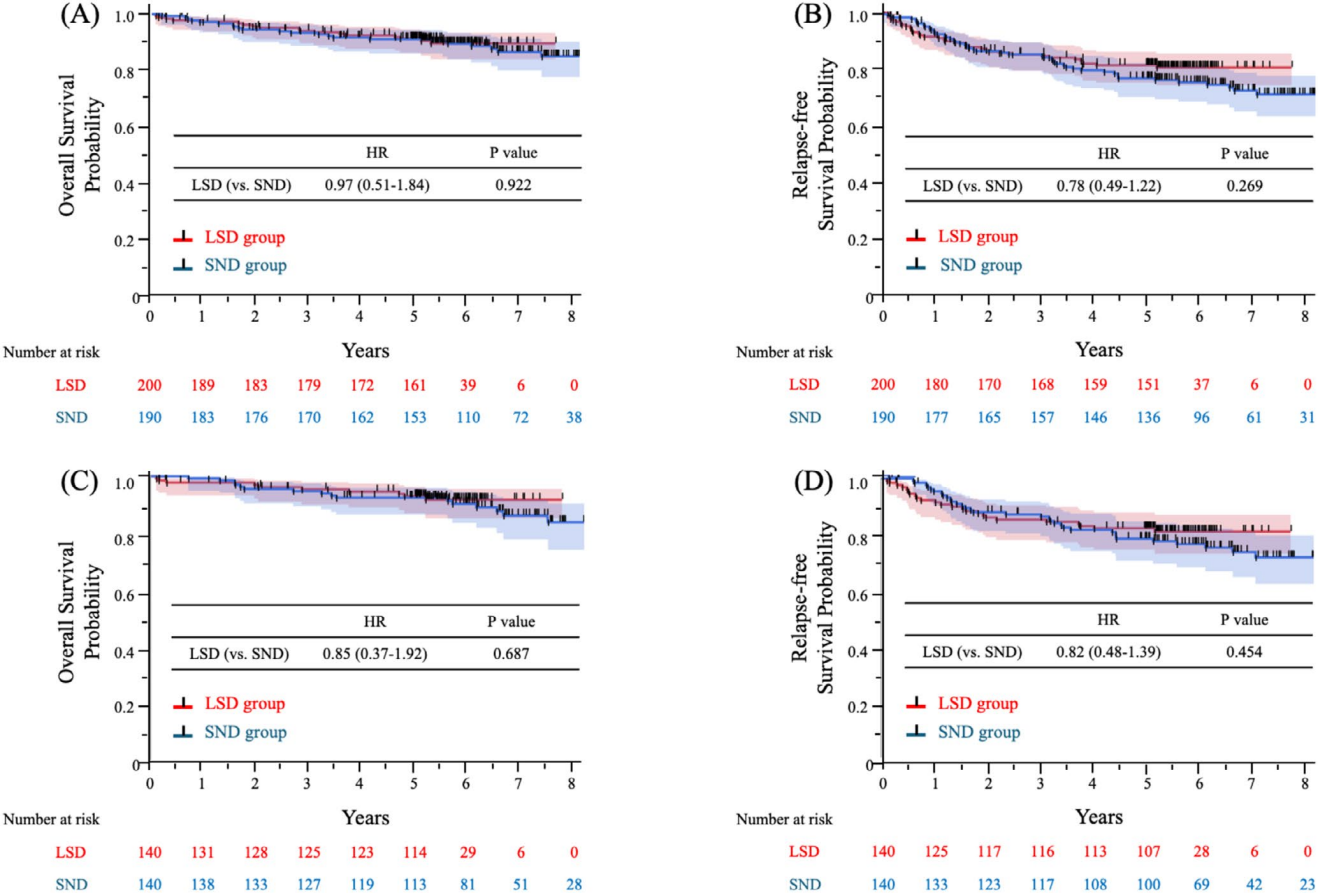
Kaplan–Meier curves for overall survival (OS) and relapse‐free survival (RFS). (A) Overall survival before propensity score matching. (B) Relapse‐free survival before propensity score matching. (C) Overall survival after propensity score matching. (D) Relapse‐free survival after propensity score matching. Overall survival and relapse‐free survival were comparable between the LSD and SND groups both before and after propensity score matching. *p*‐values were calculated using the log‐rank test.

Before PSM, the median follow‐up period for censored cases was 64.5 months (range, 0.7–97.0 months) in the LSD group and 76.9 months (range, 0.4–147.2 months) in the SND group. There were no significant differences in OS (Figure 2A) or RFS (Figure 2B) between the two groups (HR: 0.97; 95% CI: 0.51–1.84 for OS, and HR: 0.78; 95% CI: 0.49–1.22 for RFS). Five‐year OS was 91.5% in the LSD group vs. 91.3% in the SND group, and five‐year RFS was 82.9% in the LSD group vs. 78.4% in the SND group.

After PSM, 140 patients were included in each group, and the median follow‐up period for censored cases was 64.6 months (range, 0.7–93.0 months) in the LSD group and 76.2 months (range, 0.4–147.2 months) in the SND group. OS (Figure 2C) and RFS (Figure 2D) were comparable between the LSD and SND groups (HR: 0.85; 95% CI: 0.37–1.92 for OS, and HR: 0.82; 95% CI: 0.48–1.39 for RFS). Five‐year OS was 93.1% in the LSD group vs. 92.6% in the SND group, and five‐year RFS was 82.9% in the LSD group vs. 79.3% in the SND group. As a sensitivity analysis, RMST was calculated up to 60 months. The 60‐month RMST for OS was 57.5 months in the SND group and 57.6 months in the LSD group (difference, −0.06 months; 95% CI, −2.37 to 2.25; *p* = 0.962). Similarly, the 60‐month RMST for RFS was 53.1 months in the SND group and 52.3 months in the LSD group (difference, 0.85 months; 95% CI, −3.03 to 4.73; *p* = 0.667). Subgroup analyses according to tumor location were performed in the propensity score‐matched cohort. OS and RFS were comparable between the LSD and SND groups in both upper‐lobe and lower‐lobe tumors (Figure S1).

### 3.5 Pattern of Recurrence

Patterns of recurrence after PSM are summarized in Table 5. There were no significant differences between the LSD and SND groups in the overall recurrence rate. The first recurrent site (ipsilateral LNs, ipsilateral LN and other sites, or other sites) did not differ between the two groups. Causes of death were also comparable between the two groups. Regarding the ipsilateral LNs recurrences, the LSD group had six recurrences, including hilar LN recurrence in three patients, mediastinal LNs recurrence in two patients, and involvement of both sites in one patient. Among the three mediastinal LN recurrences in this group, two patients developed recurrence in LN stations that had been dissected during surgery. The remaining patient experienced combined hilar and mediastinal LNs recurrence involving both dissected and non‐dissected stations. There were 10 ipsilateral LNs recurrences in the SND group, including hilar LN recurrence in five patients, mediastinal LN recurrence in three patients, and involvement in both sites in two patients.

**TABLE 5:** Comparison of recurrence patterns between the LSD and SND groups after propensity score matching.

Variables		LSD *N* = 140 Frequency (%)	SND *N* = 140 Frequency (%)	*p*
Recurrence	Yes	19 (13.6)	29 (20.7)	0.113
The first recurrent site	Ipsilateral LNs	5 (3.6)	7 (5.0)	0.409
	Ipsilateral LNs and Others	1 (0.7)	3 (2.1)	
	Others	13 (9.3)	19 (13.6)	
Cause of death	Lung cancer	5 (3.6)	11 (7.9)	0.208
	Other diseases	5 (3.6)	4 (2.9)	
	Unknown	0 (0.0)	2 (1.4)	

Abbreviations: LN, lymph node; LSD, lobe‐specific nodal dissection; SND, systematic lymph node dissection.

## 4 Discussion

This single‐center retrospective study demonstrated that OS and RFS were comparable between the LSD and SND groups in patients with clinical stage I NSCLC, after balancing patient characteristics using PSM. Our study also showed a shorter operative time, shorter duration of chest tube placement, and shorter length of hospital stay in the LSD group. The incidence of postoperative supraventricular tachyarrhythmia was significantly lower in the LSD group. To our knowledge, this study represents one of the few investigations addressing the oncological validity of LSD under the 8th edition of the TNM stage classification and provides independent validation of previous reports, including that by Kamigaichi et al. from the National Cancer Center Hospital East in 2023 [17]. In addition, RMST analyses up to 60 months demonstrated no significant differences in either OS or RFS between the groups, supporting the robustness of our findings despite differences in follow‐up duration.

The present study showed comparable long‐term OS and RFS in patients with clinical stage I NSCLC who underwent LSD and SND after propensity score matching. According to a review article by Peng et al. five retrospective studies have compared outcomes between LSD vs. SND using propensity score‐based adjustment across different TNM editions, including one study based on the 5th edition, two based on the 7th edition, and two based on the 8th edition [19]. Among these studies, four reported no significant differences in OS or disease‐free survival (DFS) between the two groups, whereas one study demonstrated significantly better OS in patients undergoing LSD [15, 20, 21]. Hishida et al. reported improved OS in clinical stage I–II NSCLC patients who underwent LSD compared with those undergoing SND under the 7th edition of the TNM classification using an inverse probability of treatment weighting‐adjusted Cox model. The authors concluded that LSD is an alternative to SND for selected patients with c‐stage I or II NSCLC [22]. Under the 8th edition of the TNM classification, two studies compared the outcome for LSD vs. SND after adjustment for baseline characteristics. Zhao et al. reported comparable OS and DFS in patients with stage IA disease [15], while Kuroda et al. found no significant survival differences in OS and DFS in stage IA to IIIB NSCLC patients [23]. Notably, these studies did not specifically focus on patients with clinical stage I disease; therefore, our study represents an independent validation of the large‐scale propensity score‐matched cohort study reported by Kamigaichi et al. in 2023 under the 8th edition of the TNM classification [17]. Recent evidence has also suggested comparable oncologic outcomes between LSD and SND. Tanase et al. reported similar OS and RFS between the two approaches in early‐stage NSCLC, with a potential reduction in postoperative complications in the LSD group [24]. In addition, subgroup analyses according to tumor location demonstrated comparable OS and RFS between the LSD and SND groups in both upper‐lobe and lower‐lobe tumors. These findings further support the oncological validity of lobe‐specific lymph node dissection across different anatomical patterns of lymphatic drainage.

The optimal extent of LND in lung cancer surgery remains controversial. While SND has long been considered the standard approach, its survival benefit has not been consistently demonstrated, particularly in early‐stage disease [25]. Recent studies have demonstrated that lymph node metastasis patterns are lobe‐specific, providing a biological rationale for tailored LND strategies [26]. Analysis of recurrence patterns in our study further supported the oncological validity of LSD compared with SND. No significant differences were observed between the two groups in overall recurrence rates or recurrence patterns after PSM. In addition, the incidence of ipsilateral LN recurrence also did not differ between the two groups. Notably, among patients in the LSD group who developed mediastinal LN recurrence, there were no cases in which recurrence was retrospectively considered potentially preventable by SND. This finding suggests that LSD did not increase the risk of locoregional recurrence that could adversely affect long‐term outcomes, although the number of recurrence events was too small to draw definitive conclusions. In addition, pathological upstaging to stage III disease in the SND group did not include any cases in which nodal metastasis occurred exclusively outside the lobe‐specific dissection field without concurrent involvement within the LSD field. Previous studies have reported the possibility of skip mediastinal metastasis, particularly in lower‐lobe tumors in which metastasis to the upper mediastinal lymph nodes may occur without involvement of subcarinal nodes. However, in the present cohort, no cases of isolated nodal metastasis outside the LSD dissection field were identified [11], suggesting that the oncological validity of LSD was not compromised in our patient population. These findings suggest that LSD did not compromise pathological staging accuracy in this cohort.

Patients in the LSD group experienced significantly shorter operative time, less blood loss, and shorter durations of chest tube placement and postoperative hospital stay compared with those who underwent SND after PSM. Although these findings may reflect the reduced invasiveness of LSD, they should be interpreted with caution. Because of the chronological transition in our institutional surgical practice, almost all patients in the LSD group underwent VATS, whereas most patients in the SND group underwent open thoracotomy. Therefore, the surgical approach was strongly associated with both the treatment group and study period and could not be adequately adjusted for by propensity score matching. Consequently, differences in perioperative outcomes may partly reflect the influence of surgical approach rather than the extent of lymph node dissection alone.

Regarding postoperative complications, a significantly higher incidence of supraventricular tachyarrhythmia was observed in the SND group after PSM. Notably, all cases of postoperative arrhythmia occurred in patients who underwent upper mediastinal LN dissection regardless of group classification. This observation suggests that avoidance of upper mediastinal dissection in selected patients, particularly those undergoing lower lobectomies, may reduce the risk of postoperative tachyarrhythmia. In contrast, no significant differences were observed between the two groups in other postoperative complications such as prolonged air leakage, pneumonia, atelectasis, or surgical site infection, indicating a limited association between these complications and the extent of LN dissection. Interestingly, Kamigaichi et al. reported a higher incidence of postoperative arrhythmias in the SND group, whereas the incidence of other complications was comparable between the groups [17]. Our findings are consistent with these previous observations.

Several limitations of this study should be acknowledged. First, this was a retrospective, single‐center study with a relatively small sample size. Second, although PSM was applied, residual confounding cannot be completely excluded, including unmeasured factors such as detailed molecular profiling of the tumors. Third, this comparative approach has the inherent limitation that the two techniques were performed during different time periods. In particular, almost all patients in the LSD group underwent VATS, whereas most patients in the SND group underwent open thoracotomy. Therefore, differences in perioperative outcomes may have been influenced not only by the extent of lymph node dissection but also by era‐related changes in surgical approach and perioperative management. Although the choice of procedure was determined uniformly by institutional policy rather than individual surgeon preference, residual confounding related to treatment era cannot be completely excluded. Importantly, the ongoing multicenter randomized controlled trial JCOG1413, which directly compares lobe‐specific and systematic lymph node dissection in clinical stage I and II NSCLC, is expected to provide higher‐level evidence regarding the optimal extent of lymph node dissection [27].

In conclusion, this propensity score‐matched analysis based on the 8th edition of the TNM classification demonstrated that OS, RFS, and the incidence of recurrence in the LSD group were comparable to those of SND in clinical stage I NSCLC patients who underwent lobectomy with LND. These results were accompanied by a reduced incidence of supraventricular tachyarrhythmia in the LSD group compared with the SND group. In line with several previous reports, the results of this study support LSD as an oncologically acceptable and less invasive alternative to SND in contemporary surgical practice for clinical stage I NSCLC. However, the oncological validity of LSD should ultimately be confirmed in prospective multicenter studies.

## Author Contributions

**Tatsuaki Watanabe:** investigation, writing – review and editing, data curation. **Satoshi Kamata:** data curation, writing – review and editing. **Takashi Hirama:** data curation, formal analysis, supervision, investigation, writing – review and editing. **Ken Onodera:** conceptualization, investigation, methodology, data curation, writing – original draft. **Yui Watanabe:** data curation, writing – review and editing, investigation. **Toru Kawakami:** writing – review and editing, data curation. **Hirotsugu Notsuda:** conceptualization, methodology, data curation, investigation, writing – original draft. **Hisashi Oishi:** investigation, data curation, writing – review and editing. **Hiromichi Niikawa:** data curation, writing – review and editing. **Takaya Suzuki:** data curation, investigation, writing – review and editing. **Yoshinori Okada:** conceptualization, writing – review and editing, supervision. **Sakiko Kumata:** data curation, investigation, writing – review and editing.

## Funding

The authors have nothing to report.

## Ethics Statement

This study was approved by the Ethics Committee of Tohoku University Graduate School of Medicine (No. 2021‐1‐912‐1).

## Consent

The requirement for written informed consent was waived owing to the retrospective nature of the study.

## Conflicts of Interest

The authors declare no conflicts of interest.

## Supporting information


**Figure S1:** Kaplan–Meier curves for overall survival (OS) and relapse‐free survival (RFS) according to tumor location in the propensity score–matched cohort.
(A) Overall survival in upper‐lobe tumors.
(B) Relapse‐free survival in upper‐lobe tumors.
(C) Overall survival in lower‐lobe tumors.
(D) Relapse‐free survival in lower‐lobe tumors.
No significant differences in OS or RFS were observed between the LSD and SND groups in either upper‐lobe or lower‐lobe tumors.

**Table S1:** Extent of mediastinal nodal dissection during LSD and SND.
**Table S2:** Comparison of Surgical and postoperative outcomes between the LSD and SND groups.
**Table S3:** Comparison of postoperative complications between the LSD and SND.

## Data Availability

The data that support the findings of this study are available from the corresponding author upon reasonable request.
