# Supplementary figures and images for: Lobe‐Specific Versus Systematic Lymph Node Dissection in Clinical Stage I Non‐Small Cell Lung Cancer: A Propensity Score‐Matched Analysis Based on the 8th Edition of the TNM Stage Classification
Source: Thorac Cancer. 2026 Jun 29;17(13):e70337. doi: 10.1111/1759-7714.70337 (PMC13314383; doi:10.1111/1759-7714.70337)

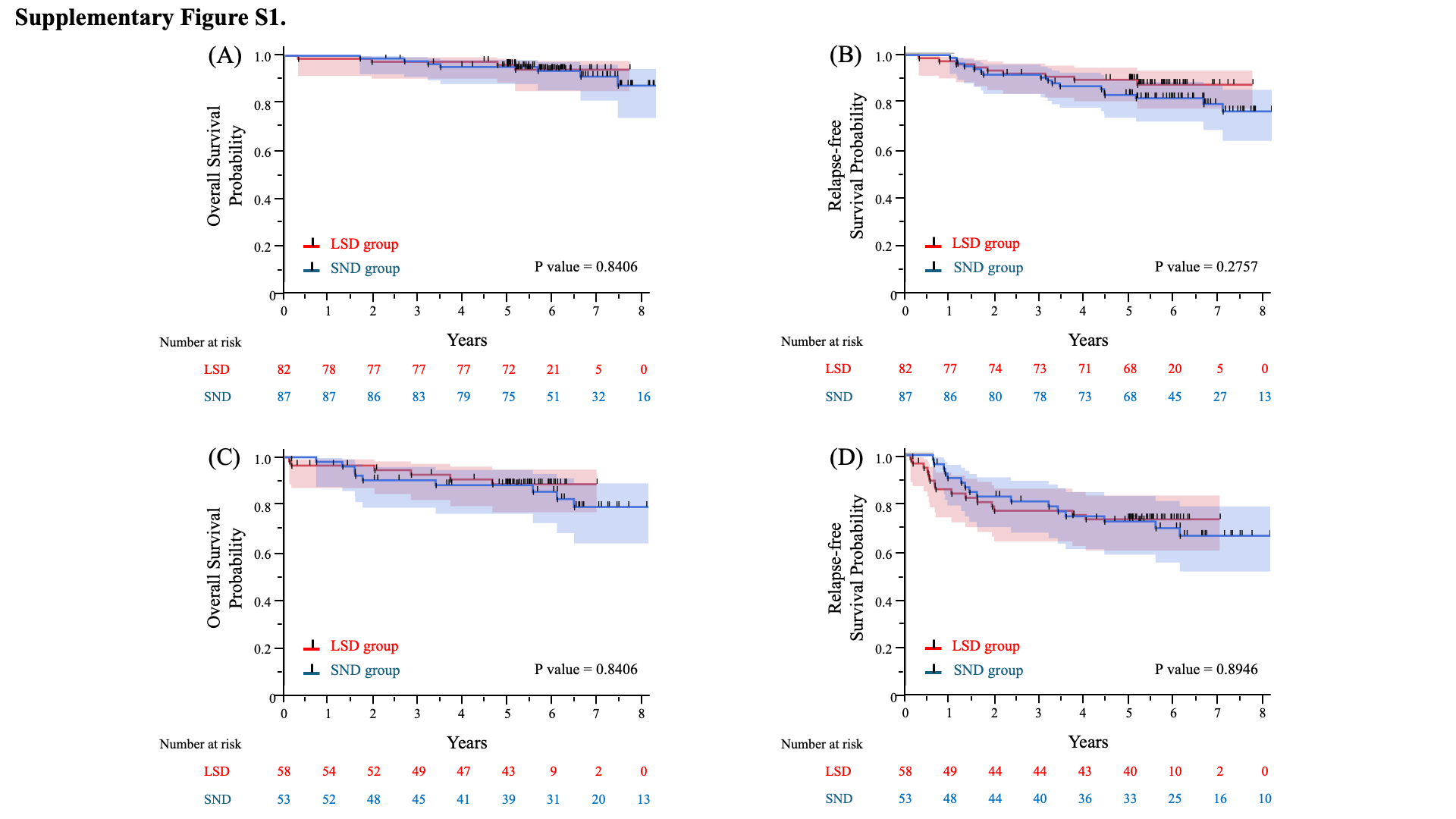

Supplement: Supplementary file 1 — Figure S1: Kaplan–Meier curves for overall survival (OS) and relapse‐free survival (RFS) according to tumor location in the propensity score–matched cohort. (A) Overall survival in upper‐lobe tumors. (B) Relapse‐free survival in upper‐lobe tumors. (C) Overall survival in lower‐lobe tumors. (D) Relapse‐free survival in lower‐lobe tumors. No significant differences in OS or RFS were observed between the LSD and SND groups in either upper‐lobe or lower‐lobe tumors. [file TCA-17-e70337-s001.tiff]
