# Supplementary material for: Lobe‐Specific Versus Systematic Lymph Node Dissection in Clinical Stage I Non‐Small Cell Lung Cancer: A Propensity Score‐Matched Analysis Based on the 8th Edition of the TNM Stage Classification
Source: Thorac Cancer. 2026 Jun 29;17(13):e70337. doi: 10.1111/1759-7714.70337 (PMC13314383; doi:10.1111/1759-7714.70337)
Supplement: Supplementary file 2 — Table S1: Extent of mediastinal nodal dissection during LSD and SND. Table S2: Comparison of Surgical and postoperative outcomes between the LSD and SND groups. Table S3: Comparison of postoperative complications between the LSD and SND. [file TCA-17-e70337-s002.docx]

Supplementary Table S1. Extent of mediastinal nodal dissection during LSD and SND

|  | Tumor location | | | |
| --- | --- | --- | --- | --- |
|  | Right upper lobe | Right lower lobe | *Left upper lobe | Left lower lobe |
| LSD | #2R, #4R | #7, #8, #9 | #4L, #5, #6 | #7, #8, #9 |
| SND | #2R, #4R, #7, #8, #9 | | #4L, #5, #6, #7, #8, #9 | |

*Except for left lingular segment

Abbreviations: **LSD, lobe-specific nodal dissection; SND, systematic lymph node dissection**

Supplementary table S2. Comparison of Surgical and postoperative outcomes between the LSD and SND groups

| Variables | LSD N=200 Median (IQR) | SND N=190 Median (IQR) | P-Value |
| --- | --- | --- | --- |
| Operative time (min) | 178 (145–213) | 224 (190–267) | <0.001 |
| Blood loss (mL) | 11 (5–42) | 42 (13–100) | 0.047 |
| Chest tube placement (days) | 2 (2–3) | 3 (2–4) | 0.004 |
| Postoperative length of hospital stay (days) | 8 (6–10) | 11 (10–15) | <0.001 |

Abbreviations: **LSD, lobe-specific nodal dissection; SND, systematic lymph node dissection**; IQR, interquartile range

Supplementary Table S3. Comparison of postoperative complications between the LSD and SND

| Variables | LSD N=200 Frequency (%) | SND N=190 Frequency (%) | P-Value |
| --- | --- | --- | --- |
| Postoperative complications | 28 (14.0) | 41 (21.6) | 0.049 |
| Arrhythmia | 2 (1.0) | 16 (8.4) | <0.001 |
| Pneumonia/Atelectasis | 4 (2.0) | 8 (4.2) | 0.206 |
| Postoperative air leakage ≧7days | 14 (7.0) | 15 (7.4) | 0.736 |
| Others | 11 (5.5) | 18 (9.4) | 0.135 |

Abbreviations: **LSD, lobe-specific nodal dissection; SND, systematic lymph node dissection**
